# dbPEC: a comprehensive literature-based database for preeclampsia related genes and phenotypes

**DOI:** 10.1093/database/baw006

**Published:** 2016-03-05

**Authors:** Alper Uzun, Elizabeth W. Triche, Jessica Schuster, Andrew T. Dewan, James F. Padbury

**Affiliations:** ^1^Department of Pediatrics, Women & Infants Hospital of Rhode Island, Providence, RI, USA; ^2^Department of Pediatrics, Brown Alpert Medical School, Providence, RI, USA; ^3^The Mandell Center for Multiple Sclerosis, Mount Sinai Rehabilitation Hospital, Hartford, CT, USA; ^4^Department of Epidemiology and Public Health, Yale University, New Haven, CT, USA; ^5^Center for Computational Molecular Biology, Brown University, Providence, RI, USA

## Abstract

Preeclampsia is one of the most common causes of fetal and maternal morbidity and mortality in the world. We built a *Database for Preeclampsia* (dbPEC) consisting of the clinical features, concurrent conditions, published literature and genes associated with Preeclampsia. We included gene sets associated with severity, concurrent conditions, tissue sources and networks. The published scientific literature is the primary repository for all information documenting human disease. We used semantic data mining to retrieve and extract the articles pertaining to preeclampsia-associated genes and performed manual curation. We deposited the articles, genes, preeclampsia phenotypes and other supporting information into the dbPEC. It is publicly available and freely accessible. Previously, we developed a database for preterm birth (dbPTB) using a similar approach. Using the gene sets in dbPTB, we were able to successfully analyze a genome-wide study of preterm birth including 4000 women and children. We identified important genes and pathways associated with preterm birth that were not otherwise demonstrable using genome-wide approaches. dbPEC serves not only as a resources for genes and articles associated with preeclampsia, it is a robust source of gene sets to analyze a wide range of high-throughput data for gene set enrichment analysis.

**Database URL**: http://ptbdb.cs.brown.edu/dbpec/

## Introduction

Preeclampsia is a multi-system hypertensive disorder of pregnancy, which complicates 2–8% of US deliveries (1, 2). It remains a major cause of maternal and fetal morbidity and mortality (2). Preeclampsia is a multifactorial disorder associated with several different clinical descriptions or ‘phenotypes’ (1). Many clinicians believe there is a difference between mild and severe or early and late preeclampsia.^3^ To date, however, there is limited evidence whether they represent different genetic ideologies.

The evidence that preeclampsia derives from both genetic and an environmental cause is based on family and epidemiological studies. Twin studies have shown that the heritability of preeclampsia is between 22% and 52% (1). A significant role for genetics in the development of preeclampsia is also supported by > 100 family studies in different populations that have reported 2- to 5-fold increased risks of preeclampsia among family members of affected women (3). The recurrence risk for preeclampsia in the daughters of preeclamptic patients is 20–40%. Epidemiologic data suggest that preeclampsia has contributions from both the maternal and fetal genome (4).

Candidate gene and genome wide studies have advantages, but also weaknesses (1). Candidate gene studies lack reproducibility that undermines the reliability of association with preeclampsia. Statistical power can be lost because of poorly identified phenotypes even though genetic effects may be strongly associated with a certain phenotype. Genome-wide association studies (GWAS) query the genome in a hypothesis-free, unbiased approach, with the potential for identifying novel genetic variants. However, the large numbers of anonymous single nucleotide polymorphisms (SNP) or copy number variations severely limits power and makes it nearly impossible, computationally, to examine combinatorial interactions. Since preeclampsia is a multifactorial disease with several phenotypes, it is important to identify specific genes associated with these different clinical descriptions. It is also important to link tissue sources in gene expression studies and genes of origin whether maternal or fetal.

The published scientific literature is the primary repository for all information documenting human disease. Semantic data mining approaches that are effective and efficient can be used for retrieval and extraction of information from the literature. We aimed to identify genes and gene variants associating with preeclampsia by using bioinformatics and computational methods. We developed a web-based, semantic data mining and aggregation tool to ‘filter’ published literature for evidence of association of preeclampsia with genes, genetic variants, SNPs or changes in gene expression. A trained curation team extracted gene and protein information from published articles specific to preeclampsia with high inter-observer reliability (5). The identified genes and sets of genes are now deposited into a user-friendly, searchable database, the *Database for Preeclampsia* (dbPEC)*.* We developed specific gene sets, reflecting tissue source and patient (maternal vs. fetal) to reduce genetic heterogeneity and more discretely associate the genetic structure of preeclampsia with its phenotypic characteristics. This extensive resource is much more than a literary record of cases and scientific experiments; it is a vast database that can be used to mine large high throughput datasets.

## Materials and Methods

**Figure 1:**
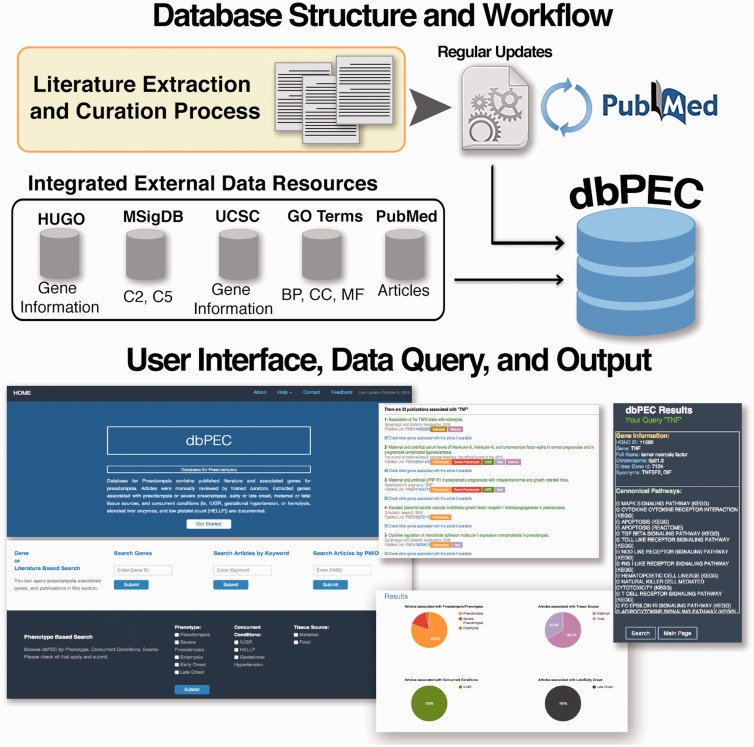
Database structure and workflow of the database for preeclampsia.

dbPEC is a relational database built on MySQL server. The web application runs on an Apache version 2.2.16 server; in-house developed PHP scripts provide data retrieval. The web interface is based on PHP and CSS scripts. The dbPEC is publicly available at http://ptbdb.cs.brown.edu/dbpec/. The workflow and design of the database is presented in Figure 1. Gene information was collected from HUGO Gene Nomenclature Committee (HGNC) database and UCSC Table Browser (6, 7). Canonical Pathways (Biocarta, KEGG and Reactome based) and GO terms were collected from Broad Institute’s Molecular Signature Database (MSigDB v5.0), respectively Collection 2 (C2) and collection 5 (C5) (8). Article information is collected from PubMed. Biyearly updates are being carried out on the database. dbPEC enables several ways to access data.

**a) Gene or literature-based search:** This section consists of three independent searchable fields.

**Search by Gene Symbol:** Users can search for specific genes by using HGNC gene symbols. A gene search returns information about preeclampsia phenotypes, concurrent conditions, tissue source, curated articles, cannonical pathways and GO terms for the gene of interest. Interactive pie charts and colored labels visually support results and helps users to navigate and filter the data. Cannonical pathways and gene ontology terms are listed and a link to each pathway’s Broad Institute MSigDB Gene Set Card is provided.

**Search articles by keyword:** Users can search the database using keywords or phrases. A keyword query will return all curated articles that have the keyword or phrase in the abstract. The results page provides detailed information about the articles and related genes identified by the query. In addition, users have the option to download gene lists and associated phenotypic characteristics as a text file.

**Search articles by PubMed identifier** **numbers:** Users can search the curated articles by PubMed Identifier numbers. If the curation team accepted the article, information about the article along with genes significantly associated with Preeclampsia are displayed.

**b)** **Phenotype-based search:** This section has three main categories formatted as check boxes: phenotypes, concurrent conditions and tissue sources. ‘Phenotype’ includes preeclampsia, severe preeclampsia, eclampsia, ‘early onset’ and ‘late onset’. ‘Concurrent conditions’ include intrauterine growth restriction (IUGR), hemolysis, elevated liver enzymes, and low platelet count (HELLP) and gestational hypertension (GH). ‘Tissue source’ specifies the tissue source for the accepted genetic data from each report. Tissue specificity was carefully curated. Definitions used are detailed in the accompanying documentation but includes: ‘maternal’ (intervillous space, basal plate, myometrium, peripheral maternal blood or chorio-decidual blood) and ‘fetal’ (placenta, amnion, umbilical arteries/veins, umbilical vein endothelial cells). Users can browse dbPEC by checking any or all options. Depending on the user’s selection, the query returns a list of genes for which there is at least one curated article that reported significant association between that gene and selected criteria. From the ‘available link’ more information about curated articles can be retrieved. We also implemented a browse all genes button into the main page that users can browse all curated genes at dbPEC.

Additional information is included on the dbPEC website under the help section as ‘Frequently Asked Questions’ and ‘User Guide’. The ‘User Guide’ can be downloaded as a PDF document. Curation is an ongoing process, so as new articles are curated and genes accepted, they are added to dbPEC. The database will be updated with newly curated data on a regular basis. Thus far, we have excluded expression array datasets, GWAS and other high throughput screening articles in our curation. Data retrieved from such sources will be deposited in future updates.

## Results

### Curation results

Approximately 2500 articles were identified using semantic data mining and natural language processing as having potential genetic associations with preeclampsia-related phenotypes from >32 000 articles in PubMed. A trained team of curators manually curated these articles and 899 of the articles were found contain ‘statistically significant’ associations between 602 genes and various preeclampsia-related phenotypes. If a statistically significant association was found, then the gene(s) and associated phenotype, concurrent conditions and tissue source, along with the article information were deposited into dbPEC. The current compilation is a literature-based collection.

### Database functionality

This database was designed to create a parsimonious set of well validated reports and genes related to preeclampsia. One of the shortcomings of high throughput technologies is the need for correction for multiple comparisons. This severely limits power and the ability to identify significant genetic associations. We previously built a database for preterm birth using a similar data mining and curation approach, dbPTB (9). Using the highly discretized gene sets from dbPTB to analyze a large GWAS, we were able to identify significant networks and pathways related to preterm birth not previously recognized (10). The gene sets for preeclampsia retrievable from this database will support similar targeted analyses of existing large data sets. We emphasize the importance of phenotype and concurrent condition-based searches when users create custom gene sets. In order to facilitate such queries our database was designed to support discreet segregation of preeclampsia phenotypes (e.g. early vs. late vs. severe) as well as concurrent conditions like HELLP or IUGR. The extracted gene sets can be used subsequently as ‘filters’ for gene set enrichment analysis, candidate gene analysis or other analyses of publically available, high throughput data sets including microarray date, SNP genotyping data or sequence data, for example in dbGAP or the Gene Expression Omnibus database. In addition, the dynamic nature of the links of the queried pages can also be used to link dbPEC pages to other online resources.

## Discussion

In 2014, we published the details of our curation process and cluster maps of the genes associated with preeclampsia phenotypes and concurrent conditions (5). We have now built a publicly accessible database to retrieve those preeclampsia-associated genes and the research articles that reported these associations. dbPEC serves as a robust and regularly updated resource of assembled preeclampsia phenotypes and associated genes. We designed the user interface of dbPEC for easy navigation and the results pages are visually supported by color coded labels and charts. We believe the dbPEC will serve investigators and clinicians as a resource where they can find information about user-defined genes of interest and/or where they can create gene sets based on preeclampsia phenotype, concurrent conditions and tissue source. Investigator chosen gene sets can be used in the analysis of sequence data, genotyping projects or for meta-analytical approaches for contrasting and combining results from different studies. There are other databases using similar bioinformatics approaches for other diseases. For preeclampsia there is currently another database, PESNPdb (http://bejerano.stanford.edu/pesnpdb/public/html/). PESNPdb is a compilation of preeclampsia-associated SNPs and related study details (11). As such, it provides complementary but different information from dbPEC.

## Conclusion

Building new tools and disease-specific databases can support interrogation of complex diseases and hasten improvements in human health. dbPEC was designed not only as a database but also as a tool to evaluate and analyze large datasets related to preeclampsia.

## Funding

This work was supported by following grants from the NIH: 1R21HD070177, 5T35HL094308, P20 RR018728 and P20GM103537. Funding for open access charge: Department resources.

*Conflict of interest*. Dr Triche reports grants from NIH Grant: 1R21HD070177, during the conduct of the study; personal fees from rEVO Biologics, Inc., outside the submitted work.
